# Deep learning-based classification with improved time resolution for physical activities of children

**DOI:** 10.7717/peerj.5764

**Published:** 2018-10-19

**Authors:** Yongwon Jang, Seunghwan Kim, Kiseong Kim, Doheon Lee

**Affiliations:** 1 Department of Bio and Brain Engineering, Korea Advanced Institute of Science & Technology (KAIST), Daejeon, South Korea; 2 Bio-medical IT Research Department, Electronics and Telecommunications Research Institute (ETRI), Daejeon, South Korea; 3 BioBrain Inc., Daejeon, South Korea

**Keywords:** Physical activity, Children, Classification, Convolutional neural network, Time resolution

## Abstract

**Background:**

The proportion of overweight and obese people has increased tremendously in a short period, culminating in a worldwide trend of obesity that is reaching epidemic proportions. Overweight and obesity are serious issues, especially with regard to children. This is because obese children have twice the risk of becoming obese as adults, as compared to non-obese children. Nowadays, many methods for maintaining a caloric balance exist; however, these methods are not applicable to children. In this study, a new approach for helping children monitor their activities using a convolutional neural network (CNN) is proposed, which is applicable for real-time scenarios requiring high accuracy.

**Methods:**

A total of 136 participants (86 boys and 50 girls), aged between 8.5 years and 12.5 years (mean 10.5, standard deviation 1.1), took part in this study. The participants performed various movement while wearing custom-made three-axis accelerometer modules around their waists. The data acquired by the accelerometer module was preprocessed by dividing them into small sets (128 sample points for 2.8 s). Approximately 183,600 data samples were used by the developed CNN for learning to classify ten physical activities : slow walking, fast walking, slow running, fast running, walking up the stairs, walking down the stairs, jumping rope, standing up, sitting down, and remaining still.

**Results:**

The developed CNN classified the ten activities with an overall accuracy of 81.2%. When similar activities were merged, leading to seven merged activities, the CNN classified activities with an overall accuracy of 91.1%. Activity merging also improved performance indicators, for the maximum case of 66.4% in recall, 48.5% in precision, and 57.4% in f1 score . The developed CNN classifier was compared to conventional machine learning algorithms such as the support vector machine, decision tree, and k-nearest neighbor algorithms, and the proposed CNN classifier performed the best: CNN (81.2%) > SVM (64.8%) > DT (63.9%) > kNN (55.4%) (for ten activities); CNN (91.1%) > SVM (74.4%) > DT (73.2%) > kNN (65.3%) (for the merged seven activities).

**Discussion:**

The developed algorithm distinguished physical activities with improved time resolution using short-time acceleration signals from the physical activities performed by children. This study involved algorithm development, participant recruitment, IRB approval, custom-design of a data acquisition module, and data collection. The self-selected moving speeds for walking and running (slow and fast) and the structure of staircase degraded the performance of the algorithm. However, after similar activities were merged, the effects caused by the self-selection of speed were reduced. The experimental results show that the proposed algorithm performed better than conventional algorithms. Owing to its simplicity, the proposed algorithm could be applied to real-time applicaitons.

## Introduction

The World Health Organization has stated that the obese population worldwide has more than doubled since 1980. In 2014, over 1.9 billion adults (over the age of 18) were overweight and more than 6 million from that number were obese (World Health Organization, 2014). The proportion of overweight and obese individuals has increased tremendously in a short period, creating a worldwide obesity trend reaching epidemic proportions. Overweight and obese conditions are especially critical in childhood. The risk of adult obesity is at least doubled in obese children relative to non-obese children, and increases for children at higher obesity levels and older ages (Serdula et al., 1993). The United States government, considering these facts seriously, established a task force to address childhood obesity. In 2010, the “Let’s Move!” campaign was launched with the first lady, Michelle Obama, playing a leading role, illustrating how significant the problem of childhood obesity has become (The White House, 2010). Overweight and obese conditions are caused by an imbalance between caloric intake and output, because excessive calorie consumption results in weight gain (Food and Nutrition Information Center, 2015). If people balance their caloric intakes and outputs, they can manage weight gain, preventing obesity. Balancing calories prioritizes knowing how many calories are consumed versus how many are expended.

Many solutions for establishing a caloric balance have been released, especially in the form of smartphone applications and wearable smart devices (Becker, 2017; Jessica Timmons, 2017). Although smartphone applications for assessing calorie intake exist, logging what and how much has been eaten to verify caloric intakes, these are not ready for common use (Gilhooly, 2017). Because human memories have both imperfect and subjective characteristics, discrepancies exist between self-reported and actual caloric intakes, with self-reported numbers normally lower than actual ones (Lichtman et al., 1992). The field of caloric intake assessment thus remains a challenging area. On the other hand, caloric expenditure assessments have been the target of much prior research (Madden, Mulrooney & Shah, 2016) and numerous commercial devices (Stables, 2017). Prior studies have primarily focused on assessing energy expenditures using accelerometers and a unit to substitute “calorie,” namely, “count” (Plasqui, 2017), analyzing caloric consumption from the perspective of overall acceleration signals generated by body movements. This simple method remains the basis for contemporary wearable calorie trackers. These solutions, however, are almost exclusively for adults. Various wearable devices (i.e., Fitbit and Jawbone) specify that users should be beyond their teenage years. Furthermore, smartphones and other smart devices are not commonly used by children. A new solution is thus required to prevent child obesity.

The resting metabolic rate (RMR) of children differs from that of adults because different factors affect RMRs in youth, such as growth, puberty, and body mass differences (Harrell et al., 2005). Children prior to or currently experiencing growth and puberty have highly changeable body masses and may display significant RMR variations due to differing growth rates and deviations between individuals (which are significantly higher than those in adults). These factors make it difficult to accurately estimate caloric consumptions for children using with statistical methods. A recent study measured energy expenditures in children during physical activities using commercialized devices, and produced results showing that energy expenditures measured with consumer activity monitors varied significantly from the reference device (Cosmed K4b^2^) and displayed wide standard deviation ranges (Blythe et al., 2017). A new approach, which is more appropriate for children than the estimation of energy expenditure during daily life, is thus required. One proposed alternative, which involves specifying the composition of body movements using physical activity classification in daily life, could provide a method for this assessment.

The word “continual” is the most critical for characterizing physical movement patterns in children. It has been empirically determined that children are active in a “continual” way. They more likely repeat “act and stop” movement patterns consistently than maintain continuous activity without stops. This tendency makes developing calorie trackers for children difficult because calories must be calculated physiologically over a sufficient timespan for determining the steady-state metabolism.

Numerous previous studies have investigated physical activity recognition based on accelerometer systems, each characterized by the aspect of its accelerometer positioning, target activities for detection, and classification algorithm. For the first characteristic, individual studies have considered multiple accelerometer positions and varying numbers of accelerometers. Some studies placed accelerometers on single body parts such as the waist (Gupta & Dallas, 2014; Karantonis et al., 2006; Mathie et al., 2004), wrist (Garcia-Ceja et al., 2014; Yang, Wang & Chen, 2008), or trunk (Massé et al., 2015), whereas others attached sensors to multiple body parts such as the thighs, necklaces, and wrists (Pirttikangas, Fujinami & Nakajima, 2006); wrists, chests, and hips (Moncada-Torres et al., 2014); thighs, waists, chests, and ankles (Gupta & Dallas, 2014); chests, thighs, and ankles (Chamroukhi et al., 2013; Moncada-Torres et al., 2014); and chests, waists, thighs, and sides (Gao, Bourke & Nelson, 2014). For target activities, some studies detected primarily simple postures (Gupta & Dallas, 2014; Mathie et al., 2004), whereas others focused on ambient movements such as walking or running (Pirttikangas, Fujinami & Nakajima, 2006; Yang, Wang & Chen, 2008), and still others covered primarily daily living activities such as eating, writing, and talking (Moncada-Torres et al., 2014; Salarian et al., 2007). These studies employed various types of classification algorithms desirable for their target activities, including supervised machine learning algorithms such as k-nearest neighbors (Foerster, Smeja & Fahrenberg, 1999; Kaghyan & Sarukhanyan, 2012), support vector machines (Anguita et al., 2013; Krause et al., 2005), random forests (Bedogni, Di Felice & Bononi, 2012), Gaussian mixture models (Mannini & Sabatini, 2010), artificial neural networks (Ong, Koseki & Palafox, 2013; Yang, Wang & Chen, 2008) and unsupervised algorithms such as K-Means (Cottone et al., 2013; Lester et al., 2005) and Hidden Markov Models (Trabelsi et al., 2013).

Unfortunately, these studies are not appropriate for application to the daily life of real children. For studies requiring more than two wearable devices (Chamroukhi et al., 2013; Gao, Bourke & Nelson, 2014; Gupta & Dallas, 2014; Moncada-Torres et al., 2014; Pirttikangas, Fujinami & Nakajima, 2006), children failed to comply with equipping devices for long periods. These studies used multiple devices in multiple body locations to measure various acceleration signals. Although strategies utilizing multiple devices could be useful for producing definitive results in this research topic, they are uncomfortable for application in daily life, especially for children. Other cases encountered issues when there are insufficient overall or desirable activities for verifying the amount of physical movement in children (Garcia-Ceja et al., 2014; Karantonis et al., 2006; Massé et al., 2015; Mathie et al., 2004). Some studies employed various sensors (Salarian et al., 2007), and highly complex algorithms (Yang, Wang & Chen, 2008); however, if the target users are children, small devices with long battery operating times and only one sensor should be used, and the processing algorithm should be fast and simple. Investigating this perspective, this study aimed to develop a new algorithm appropriate for classifying the physical activities performed by children based on a single three-axis accelerometer.

The “continual” characteristics of children affect not only the calorie measurement but also the physical activity classification. To classify short-time movements, time resolution of classifier should be sufficiently high to cover the movement time. In general, however, a shorter duration in the data to be processed corresponds to a lower classification accuracy. This phenomenon was also noted in a previous study (Trost et al., 2012). Thus, both the time resolution and accuracy must be addressed when classifier is used to evaluate the case of children.

The machine learning method is one of the best solutions for classifying physical activities, with novel deep learning method recently demonstrating outstanding performances. From these emerging methods, the convolutional neural network (CNN) was adopted in this study because of two of its merits: first, CNNs are capable of simplifying the classifier, enabling a single processing line by handling signal inputs as image files to treat opposing characteristics of real time applications when physical activities contain mixes of static and dynamic, steady-state and transient, or constant and sporadic activities; and second, CNNs facilitate high-resolution algorithms. When signals must be analyzed in the frequency domain, the time–frequency transform relationship makes time domain signal durations important. There is an incompatible relationship between signal time resolution and category classification accuracy which could be resolved by a CNN.

## Materials & Methods

### Methods overview

Developing a physical activity classification algorithm requires the acquisition of actual acceleration data from body movements of target-age children. This study formed a research plan for this data acquisition including subject recruitment, a series of physical activity protocols, and the preparation of equipment for recording physical activity data.

Acquired data were processed into an appropriate form for generating datasets, which were then used to develop an algorithm classifying the physical activities of the children. A deep machine learning method was used to ensure high algorithm performance. This deep machine learning algorithm used in conjunction with the datasets proved capable of classifying the physical activities performed children.

### Preparation of data acquisition

A total of 136 participants (86 boys and 50 girls) took part in this experiment from one school and various sports clubs. From these 136 participants, we obtained valid data for 115 subjects consisting of 75 boys and 40 girls. The data from 21 subjects were dropped owing to pre-existing physical or mental illnesses/conditions, and in some instances, the data was invalid owing to device malfunctions or unexpected noise. The ages of participants for whom the data were valid were distributed between 8.5 and 12.5 years with a mean of 10.5, and a standard deviation of 1.1.

An experimental protocol explaining the types of movements to be conducted, as well as when and how long they should last, was designed to obtain the physical activity data generated by the body movements. Informed consent was obtained from the participants as well as from their parents. The consent form explained the contents of the experiment and the protocol. The experimental plan and consent form were approved by the internal review board of the Catholic University of Korea, Seoul ST. Mary’s Hospital (KIRB-00472_9-002).

**Figure 1:**
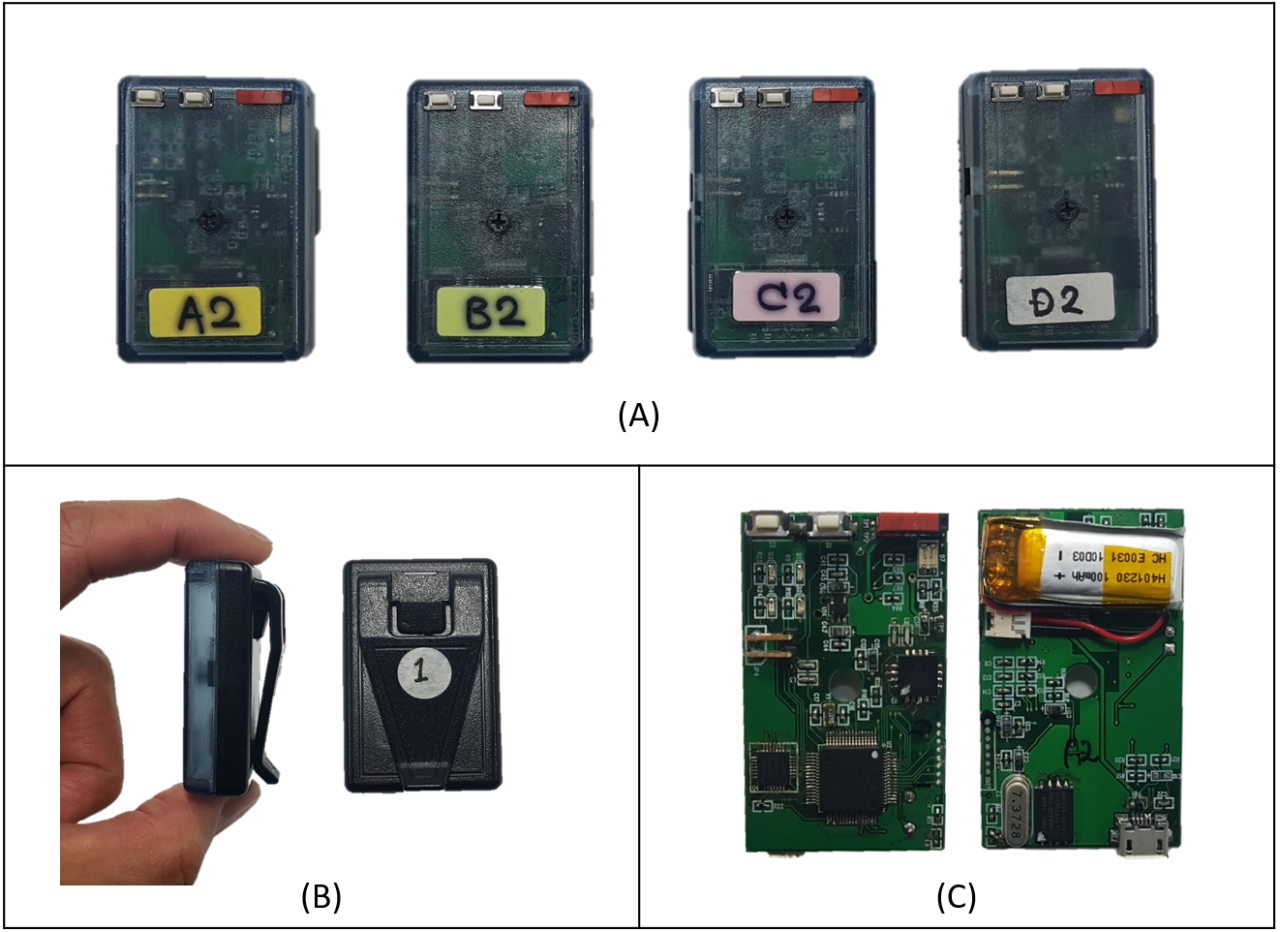
Appearance of devised data acquisition system. (A) Front face of custom –made accelerometer modules. (B) Housing with clip. (C) Front and back sides of electronics board. Photographs by Yongwon Jang.

Participants fulfilled protocols composed of various movements, including walking, running, and moving on stairs. Each individual wore an accelerometer module on their waist between the center of the belly and right pelvic, along the waistline of the pants (standard positioning of a pedometer) as they completed the protocol. The wearable data-recording module used in this study was designed as an accelerometer system to collect acceleration signals generated by body movements and fabricated from a microcontroller unit, three-axis acceleration sensor, memory chip, power unit, etc. Figure 1 shows the appearance of the devised system. The accelerometer module had a single three-axis accelerometer and no other sensors, thereby minimizing the use of sensor signals and power while maximizing the portability and convenience. The module accelerometer range was set to 4 × gravity in both positive and negative directions (±4 g). The module is 50 mm ×30 mm ×15 mm in dimension, weighs 21 g, and has a case with a clip to fasten it to the wearing point of pants.

### Data acquisition procedure

Participants followed the physical activity protocol composed of various motions including sitting down on and standing up from a chair, jumping in place, walking, running, ascending and descending stairs, and jumping rope. A table sheet with a summarized protocol listing selected physical activities was used during the experiment and the physical activity classification algorithm development. Table 1 shows the protocol of selected activities. The net protocol conduction time, excluding resting, was 22 min. Although subjects took breaks between protocol activities to catch their breath and recover their stamina, break times were not marked in the table sheet because each subject had a different recovery condition and the length of break periods varied widely. Recovery break times were based on the criterion of heart rate, such that subjects were considered to have recovered when their heart rates approached a resting level. The usual break time ranged between 2 and 5 min according to the activities.

**Table 1:** Protocol with selected activities for the experiment.

**Group**		**Physical activity**		**Net time (min)**	**Cumulative time (min)**	**Data samples**
A	1	Stay still (Sitting/Standing)		3	3	20.7 k
	2	Sitting/Standing repeat		2	5	25.5 k/25.2 k
B	1	Walking	slow	2	7	19.1 k
	2		fast	2	9	16.4 k
	3	Running	slow	2	11	16.0 k
	4		fast	2	13	17.7 k
C	1	Stairs	Ascending	2	15	14.2 k
	2		Descending	2	17	10.4 k
	3	Jumping rope		3	20	18.4 k

**Notes.**

183.6 k samples in total.

Table 1 shows three activity groups: Group A lists a still state and intermittent movements, group B lists basic and highly frequent steady-state movements (i.e., walking and running), and group C lists relatively infrequent steady-state movements (i.e., moving on stairs and jumping rope).

As the order of physical activities could influence movement patterns, the effect of ordering was considered before subjects performed the protocol. For example, successive vigorous activities could make create differences in movement relative to other activity sequences even with sufficient resting times. To circumvent this ordering effect, subjects were evenly divided into six shuffled group orderings: A-B-C, A-C-B, B-A-C, B-C-A, C-A-B, and C-B-A. Group B activities (walking and running) were repeated at slow and fast speeds determined entirely by the participants.

**Figure 2:**
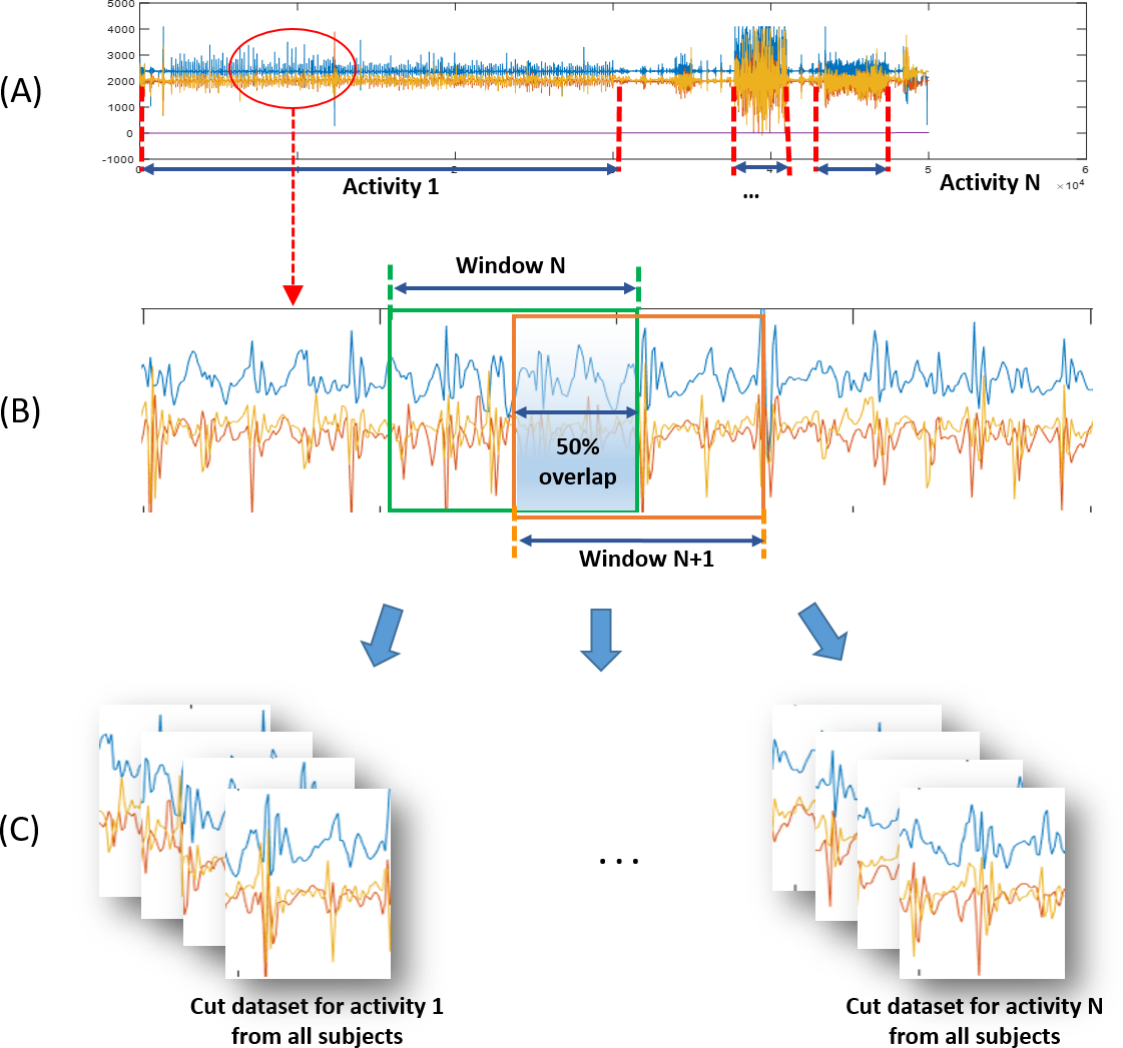
Preprocessing procedures for physical activity training of classification algorithm. (A) Acceleration signal data file of each subject. (B) Magnified portion of acceleration signals and windowing description (Window Size = 128 points = ∼2.8 s). (C) Dataset of activities from all subjects.

### Data preprocessing and learning for classification

A. Data preprocessing

(i) Data collection and filtering
  While the participants performed physical activities according to the above protocol, the accelerometers they wore measured acceleration signals that were then digitized by the microcontroller unit and stored in a mounted memory chip. Data sampling was performed at a frequency of 45.4 Hz. Stored data files containing all activity data segments for an ordered protocol (as in Fig. 2A) were then downloaded onto a PC for data processing. A high-pass frequency domain filter (over 0.5 Hz) was used to eliminate the bias signal from data.
(ii) Data samples and augmentation
  The filtered data were divided into protocol activities and cut to constant time windows with widths of 128 data points, corresponding to ∼2.8 s. At this stage, data augmentation with overlaps and rotations allowed us to enrich and diversify our data samples. Adjacent windows shared half their data with an overlap of 50%. In addition, the data was rotated at random degrees within ±10 for yawing, ±15 for pitching, and ±20 for rolling. This was used to simulate the rotated state of equipped accelerometer. Figure 2B depicts the windowing and cutting data processes.
(iii) Data preparation for learning
  The augmented window-sized data samples from all participant data (∼184,000 samples as listed in Table 1) were sorted by activity to form ten dataset types, one for each activity: slow walking (WS), fast walking (WF), slow running (RS), fast running (RF), walking up the stairs (SU), walking down the stairs (SD), jumping rope (JR), standing up (ST), sitting down (SI), and keeping still without activity (NA). Figure 2C shows the assignment of window-sized data samples to their corresponding datasets. The dataset bundles for the ten activities were then fed into the CNN training process.

B. Convolutional feature extraction

**Figure 3:**
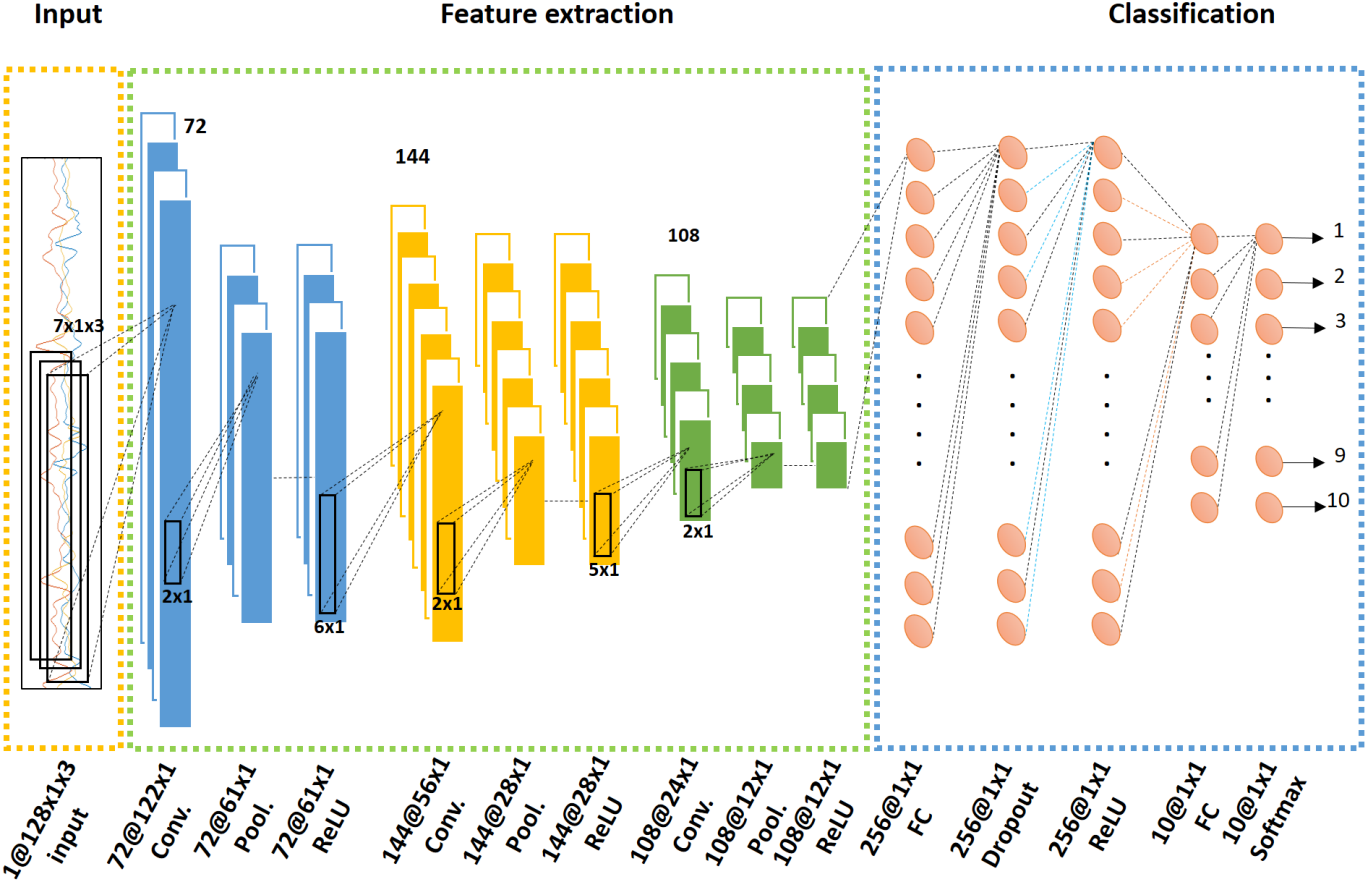
Convolutional neural network (CNN) structure with input, feature extraction, and classification stages. The feature extraction process was composed of a ternary convolution block expressed with different colors. The classification stage included fully connected layers and a dropout function, and provided an output from a softmax function.

Figure 3 shows the CNN structure and describes the training procedure. The overall network architecture was composed of an input stage, a feature extraction stage with three convolutional layer blocks, and an output provision classification stage. The input stage received the preprocessed dataset. The feature extraction stage followed a triple ternary convolution block structure including convolution, pooling, and activation functions. Each convolution block is expressed in Fig. 3 with a different color. The last classification stage consisted of a fully connected layer, dropout, and softmax operator. The final network outputs came from the softmax operation results.

(i) Input layer
  In a CNN, a layer can be represented by the general expression function **y** = ***f***(**x**) where both the input **x** and output **y** are tensors. The network input is a Height(H) × Width(W) × Dimension(D) sized tensor data. In this study, the input was the time series accelerometer data where H = window size, W = single dimension, and D = number of sensor axes. As shown in Fig. 3, this input size H × W × D was equal to 128 × 1 × 3.
(ii) Convolution layer
  The convolution layer computed the input **x** convolution using filters ***f***. The element expressions can be represented as:
  (1) \begin{eqnarray*}\mathbf{x}\in {\mathbb{R}}^{H\times W\times D},\mathbi{f}\in {\mathbb{R}}^{{H}^{{}^{{^{\prime}}}}\times {W}^{{}^{{^{\prime}}}}\times D\times {D}^{{}^{{^{\prime}}{^{\prime}}}}},\mathbf{y}\in {\mathbb{R}}^{{H}^{{}^{{^{\prime}}{^{\prime}}}}\times {W}^{{}^{{^{\prime}}{^{\prime}}}}\times {D}^{{}^{{^{\prime}}{^{\prime}}}}}.\end{eqnarray*}
  The output of this convolution layer was the three-dimensional convolution result, as represented below in Eq. (2) where *i*, *j* are spatial subscripts and *d* represents depth.
  (2) \begin{eqnarray*}{y}_{i{^{\prime\prime}}j{^{\prime\prime}}d{^{\prime\prime}}}={b}_{d{^{\prime\prime}}}+\sum _{{i}^{{^{\prime}}}=1}^{{H}^{{^{\prime}}}}\sum _{{j}^{{^{\prime}}}=1}^{{W}^{{^{\prime}}}}\sum _{{d}^{{^{\prime}}}=1}^{D}{f}_{{i}^{{^{\prime}}}{j}^{{^{\prime}}}d}\times {x}_{i{^{\prime\prime}}+{i}^{{^{\prime}}}-1,j{^{\prime\prime}}+{j}^{{^{\prime}}}-1, {d}^{{^{\prime}}},d{^{\prime\prime}}}.\end{eqnarray*}
(iii) Pooling layer
  Pooling layers are used to identify the most important features and compresses layer sizes by subsampling. In this study, a max pooling method (as in Eq. (3)) compared consecutive convolutional result values in H′ × W′ sized areas and the maximum value survived to go through to next layer. The comparison area (H′ × W′ = 2 × 1) was exclusive, decreasing the spatial resolution by the factor of area size.
  (3) \begin{eqnarray*}{y}_{i{^{\prime\prime}}j{^{\prime\prime}}d}=\max _{1\leq {i}^{{^{\prime}}}\leq {H}^{{^{\prime}}},1\leq {j}^{{^{\prime}}}\leq {W}^{{^{\prime}}}}{x}_{i{^{\prime\prime}}+{i}^{{^{\prime}}}-1,j{^{\prime\prime}}+{j}^{{^{\prime}}}-1, d}\end{eqnarray*}
(iv) Activation function
  An activation function followed the max pooling step in the convolution block. A rectified linear unit (ReLU), as expressed in Eq. (4), was selected for this function to mitigate the vanishing gradient problem and reduce learning time (Glorot, Bordes & Bengio, 2011). Any negative output values remaining after the max pooling layer were set to zero.
  (4) \begin{eqnarray*}{y}_{ijd}=\max \nolimits \{0,{x}_{ijd}\}.\end{eqnarray*}
(v) Repeated convolution blocks
  Three convolution blocks were used, combining the steps of convolution, max pooling, and ReLU. Figure 3 shows the ternary convolution block structure, the filter sizes of which were 7 × 1 × 3 × 72, 6 × 1 × 1 × 144, and 5 × 1 × 1 × 108, respectively.

C. Classification for output

(i) Fully connected layer
  The ReLU results of each layer, as with all layer outputs, were connected to the weighted nodes of the next layer. This study featured 256 empirically set nodes.
(ii) Dropout layer
  A dropout layer was imported to prevent overfitting, which may occur if a node in one layer excessively influences the subsequent layer. The dropout step dropped randomly selected nodes in fully connected layers with a set turn-on rate. Regularizing dropouts thinned the ongoing training network and created an ensemble effect within the thinned network (Hinton et al., 2012; Srivastava et al., 2014). This step could be performed in every fully connected layer except the final one for output. In this study case, only a single dropout layer was inserted in the penultimate fully connected layer using a rate of 0.5.
(iii) Softmax layer
  The softmax function was applied across feature channels to consolidate network results. This normalization method was applied to make that the sum of output probability distributions 1, as shown in Eq. (5).
  (5) \begin{eqnarray*}{y}_{ijk}= \frac{{e}^{{x}_{ijk}}}{\sum _{t=1}^{D}{e}^{{x}_{ijk}}} .\end{eqnarray*}
(iv) Classification losses
  The categorical loss function *l*(x, c) calculates the difference between a prediction x and ground truth c. The classification error *l* is zero if the predicted class with the largest score is same to ground truth, otherwise the error is 1.

D. Training and validation strategy

The stochastic gradient descent (SGD) method was used for backpropagation during the training process. The gradients for each parameter were averaged over the training cases in each batch. The learning rate started at 0.0003 and decreased by a log scale function with a factor of 0.5 for every hundred epochs. The momentum and weight decay parameters were set to 0.9 and 0.0005, respectively, and the batch size (set by experimental trials) was 128. It was determined the training epoch was optimized just before overfitting. The critical point was determined to be when the slope of the log loss curve changed from negative to positive, although the classification error continued decreasing past this point. Epoch points (such as the global minimum of the error function) were considered to be optimal points. Finally, the trained network was validated using the 10-fold cross-validation method. Ten groups of data were established such that nine groups were used for training and the remaining group was used for validation. Each group was designed to participate in validation in turn. The performance was measured using the mean of the validation results for each group.

## Results

The developed CNN algorithm classified physical activity data for every 1.4 s (with a window size of 2.8 s) into ten classes: WS, WF, RS, RF, SU, SD, JR, ST, SI, and NA. Table 2 shows the classification results in confusion matrix form. Each cell in the table represents the number of samples classified by the trained CNN algorithm. The vertical title indicates the actual class (input class of the algorithm), whereas the horizontal title indicates the classified results (output class of the algorithm). The ten colored diagonal cells indicate samples correctly classified by the trained CNN algorithm. The overall accuracy, drawn from 10-fold cross-validation, was 81.2%. The ratios of various output classifications from each class are listed in Table 3, with the correctly classified ratios, which could be expressed in terms of recall or sensitivity, located in the diagonal cells. The performance indicators of recall, precision, and f1 score are presented in Table 4 for each class. These numbers can be used for alternative algorithm assessments.

**Table 2:** The confusion matrix of classification results using the 10-fold cross-validation of the developed CNN algorithm.

Input/Output		Target class										Sum by row
		**WS**	**WF**	**RS**	**RF**	**SU**	**SD**	**JR**	**ST**	**SI**	**NA**	
Output Class	**WS**	**1,368**	428	29	5	116	19	4	0	0	2	1,971
	**WF**	357	**1,060**	52	16	43	7	10	0	0	0	1,545
	**RS**	2	47	**886**	366	42	44	59	0	0	0	1,446
	**RF**	1	5	536	**1,321**	23	40	43	0	0	0	1,969
	**SU**	136	66	8	6	**906**	64	32	2	1	0	1,221
	**SD**	33	21	51	36	65	**796**	70	2	2	0	1,076
	**JR**	10	14	38	23	193	67	**1,621**	0	0	0	1,966
	**ST**	4	1	0	0	6	1	0	**2,425**	66	6	2,509
	**SI**	1	1	1	0	1	3	0	73	**2,468**	8	2,556
	**NA**	3	1	0	0	21	0	0	13	13	**2,053**	2,104
Sum by column		1,915	1,644	1,601	1,773	1,416	1,041	1,839	2,515	2,550	2,069	18,363

**Notes.**

The number in table mean classified cases with the trained CNN algorithm. For example, 1,368 samples were classified as WS from 1915 WS input samples.

**Table 3:** The confusion matrix of the trained network for each class.

Input/Output		Target Class									
		**WS**	**WF**	**RS**	**RF**	**SU**	**SD**	**JR**	**ST**	**SI**	**NA**
Output class	**WS**	**71.4**	**26.0**	1.8	0.3	8.2	1.8	0.2	0.0	0.0	0.1
	**WF**	**18.6**	**64.5**	3.3	0.9	3.0	0.7	0.5	0.0	0.0	0.0
	**RS**	0.1	2.9	**55.3**	20.6	3.0	4.2	3.2	0.0	0.0	0.0
	**RF**	0.1	0.3	33.5	**74.5**	1.6	3.8	2.3	0.0	0.0	0.0
	**SU**	7.1	4.0	0.5	0.3	**64.0**	6.2	1.7	0.1	0.0	0.0
	**SD**	1.7	1.3	3.2	2.0	4.6	**76.5**	3.8	0.1	0.1	0.0
	**JR**	0.5	0.9	2.4	1.3	13.6	6.4	**88.2**	0.0	0.0	0.0
	**ST**	0.2	0.1	0.0	0.0	0.4	0.1	0.0	**96.4**	2.6	0.3
	**SI**	0.1	0.1	0.1	0.0	0.1	0.3	0.0	2.9	**96.8**	0.4
	**NA**	0.2	0.1	0.0	0.0	1.5	0.0	0.0	0.5	0.5	**99.2**
Sum by column		100	100	100	100	100	100	100	100	100	100

**Notes.**

Numbers in the cells are expressed in %. Each column makes 100% in total.

**Table 4:** The various performance indicators of developed algorithm for each class.

Class/Indicators	WS	WF	RS	RF	SU	SD	JR	ST	SI	NA
Recall	0.714	0.645	0.553	0.745	0.640	0.765	0.882	0.964	0.968	0.992
	±0.020	±0.023	±0.024	±0.020	±0.025	±0.026	±0.015	±0.007	±0.007	±0.004
Precision	0.694	0.686	0.613	0.671	0.742	0.740	0.825	0.967	0.966	0.976
	±0.021	±0.022	±0.024	±0.022	±0.023	±0.027	±0.017	±0.007	±0.007	±0.007
F1 score	0.704	0.665	0.582	0.706	0.687	0.752	0.852	0.965	0.967	0.984

The recall numbers for the ten individual classes were 71.4% for WS, 64.5% for WF, 55.3% for RS, 74.5% for RF, 64.0% for SU, 76.5% for SD, 88.2% for JR, 96.4% for ST, 96.8% for SI, and 99.2% for NA. Non-ambulatory physical activity classes (ST, SI, and NA) made up a leading group which demonstrated the best performance, whereas ambulatory moving classes could be divided into a lower performance group (WS, WF, RS, RF, SU, and SD) with recall numbers under 80%. The vigorous activity class, JR, showed an accuracy of 88.2%.

The precisions of the ten individual classes, which showed similar attributes to the recall values, were 69.4% for WS, 68.6% for WF, 61.3% for RS, 67.1% for RF, 74.2% for SU, 74.0% for SD, 82.5% for JR, 96.7% for ST, 96.6% for SI, and 97.6% for NA. The recall leading group also demonstrated high precision ratios (>96.6%). The ambulatory classes (WS, WF, RS, RF, SU, and SD) demonstrated low performances of less than 80%; however, the differences among classes were reduced compared to the recall values. JR were ranked in the middle with a performance of 80%, the same as recall case. The f1 scores of each class, which demonstrated similar characteristic to the above indicators, are presented in Table 4. Non-ambulatory activity classes showed high f1 scores, whereas ambulatory activities show relatively low f1 scores.

The performance index of the developed algorithm differs when similar activities are combined. For this study, WS and WF (types of walking) were merged into WX, RS and RF (types of running) were merged into RX, and SU and SD (stair-based activities) were grouped into SX. This reduced the total number of classes to seven: WX, RX, SX, JR, ST, SI, and NA. Merged class results are shown in Table 2 in cells bounded by colored boxes. Table 5, the converted confusion matrix using merged class groups, shows the number of processed samples from merged classes. The distribution of correct or incorrect ratios is presented in Table 6, demonstrating the overall accuracy of 91.1%. The recall, precision, and f1 score numbers for each merged class are shown in Table 7. The recall numbers for the seven merged classes were significantly improved from the ten-class case: 90.0% for WX, 92.0% for RX, 75.0% for SX, 88.0% for JR, 96.0% for ST, 97.0% for SI, and 99.0% for NA. The precision numbers for the seven classes were 91.4% for WX, 91.0% for RX, 79.7% for SX, 82.5% for JR, 97.0% for ST, 97.0% for SI, and 97.6% for NA, whereas the f1 scores were 0.908 for WX, 0.916 for RX, 0.770 for SX, 0.852 for JR, 0.970 for ST, 0.970 for SI, and 0.984 for NA. It can thus be stated that the recall, precision, and f1 scores were improved when similar activities were merged.

**Table 5:** The converted confusion matrix with seven merged classes.

Input/Output		Target class							
	**WX**	**RX**	**SX**	**JR**	**ST**	**SI**	**NA**	Sum by row
Output class	**WX**	**3,213**	102	185	14	0	0	2	3,516
	**RX**	55	**3,109**	149	102	0	0	0	3,415
	**SX**	256	101	**1,831**	102	4	3	0	2,297
	**JR**	24	61	260	**1,621**	0	0	0	1,996
	**ST**	5	0	7	0	**2,425**	66	6	2,509
	**SI**	2	1	4	0	73	**2,468**	8	2,556
	**NA**	4	0	21	0	13	13	**2,053**	2,104
Sum by column		3,559	3,374	2,457	1,839	2,515	2,550	2,069	18,363

**Notes.**

The number in table mean classified cases with the merged classes.

**Table 6:** The converted confusion matrix with seven merged classes.

Input/Output		Target Class						
		**WX**	**RX**	**SX**	**JR**	**ST**	**SI**	**NA**
Output Class	**WX**	**90.3**	3.0	7.5	0.8	0.0	0.0	0.1
	**RX**	1.5	**92.1**	6.1	5.5	0.0	0.0	0.0
	**SX**	7.2	3.0	**74.5**	5.5	0.2	0.1	0.0
	**JR**	0.7	1.8	10.6	**88.1**	0.0	0.0	0.0
	**ST**	0.1	0.0	0.3	0.0	**96.4**	2.6	0.3
	**SI**	0.1	0.0	0.2	0.0	2.9	**96.8**	0.4
	**NA**	0.1	0.0	0.9	0.0	0.5	0.5	**99.2**
Sum by column		100	100	100	100	100	100	100

**Notes.**

The number in table mean classified cases with the merged classes.

**Table 7:** The various performance indicators of the developed algorithm with seven merged classes.

Class/Indicators	WX	RX	SX	JR	ST	SI	NA
Recall	0.900	0.920	0.750	0.880	0.960	0.970	0.990
	±0.010	±0.009	±0.017	±0.015	±0.008	±0.007	±0.004
Precision	0.914	0.910	0.797	0.825	0.970	0.970	0.976
	±0.009	±0.010	±0.016	±0.017	±0.007	±0.007	±0.007
F1 score	0.908	0.916	0.770	0.852	0.970	0.970	0.984

For proper evaluation, the developed algorithm was compared to other three well-known, conventional learning classification algorithms: support vector machine (SVM), decision tree (DT), and k-nearest neighbors (k-NN). Table 8 shows the compared overall accuracies for these and the developed CNN algorithm for ten individual classes and seven merged classes. The detail performance indicators from both cases are shown in Tables 9 and 10 for all comparison targets. The contents of all three tables indicated the merged classes case performed better than the individual classes. After class merging, the conventional algorithm used for comparison showed a performance improvement of approximately 10% like the proposed CNN.

**Table 8:** The overall accuracies of the compared target algorithms and CNN.

Classifierr/Overall accuracy	CNN	SVM	DT	k-NN
10 individual classes (%)	81.2 ± 0.6	65.3 ± 0.7	63.9 ± 0.7	55.4 ± 0.7
7 merged classes (%)	91.1 ± 0.4	74.7 ± 0.6	73.2 ± 0.6	65.3 ± 0.7

**Notes.**

The compared target algorithms showed best results under the below conditions.

SVM kernel function, Gaussian, kernel scale, 3 DT split criterion, Gini’s diversity index, maximum number of splits, 5,000 k-NN distance metric, Euclidean (weighted), number of neighbors, 10

**Table 9:** The performance indicators of the compared target algorithms with the ten individual classes.

Class/Indicators		WS	WF	RS	RF	SU	SD	JR	ST	SI	NA
SVM	Recall	0.740	0.481	0.597	0.771	0.192	0.374	0.865	0.687	0.531	0.985
		±0.020	±0.024	±0.024	±0.020	±0.021	±0.029	±0.016	±0.018	±0.019	±0.005
	Precision	0.499	0.527	0.558	0.661	0.449	0.546	0.863	0.614	0.619	0.981
		±0.022	±0.024	±0.024	±0.022	±0.026	±0.030	±0.016	±0.019	±0.019	±0.006
	F1 score	0.596	0.503	0.576	0.712	0.269	0.444	0.864	0.649	0.572	0.983
DT	Recall	0.617	0.519	0.547	0.783	0.330	0.380	0.841	0.598	0.586	0.980
		±0.022	±0.024	±0.024	±0.019	±0.025	±0.029	±0.017	±0.019	±0.019	±0.006
	Precision	0.534	0.523	0.581	0.643	0.413	0.478	0.834	0.621	0.587	0.979
		±0.022	±0.024	±0.024	±0.022	±0.026	±0.030	±0.017	±0.019	±0.019	±0.006
	F1 score	0.572	0.521	0.563	0.707	0.367	0.424	0.838	0.610	0.586	0.980
k-NN	Recall	0.497	0.374	0.556	0.752	0.207	0.264	0.818	0.492	0.438	0.944
		±0.022	±0.023	±0.024	±0.020	±0.021	±0.027	±0.018	±0.020	±0.019	±0.010
	Precision	0.411	0.423	0.552	0.650	0.276	0.422	0.826	0.504	0.463	0.772
		±0.022	±0.024	±0.024	±0.022	±0.023	±0.030	±0.017	±0.020	±0.019	±0.018
	F1 score	0.450	0.397	0.554	0.697	0.237	0.325	0.822	0.498	0.450	0.849

**Table 10:** The performance indicators of the compared target algorithms with the seven merged classes.

Class/Indicators		WX	RX	SX	JR	ST	SI	NA
SVM	Recall	0.860	0.910	0.330	0.870	0.690	0.530	0.980
		±0.011	±0.010	±0.019	±0.015	±0.018	±0.019	±0.006
	Precision	0.703	0.815	0.619	0.863	0.614	0.619	0.981
		±0.015	±0.013	±0.019	±0.016	±0.019	±0.019	0.006
	F1 score	0.773	0.862	0.432	0.864	0.649	0.572	0.983
DT	Recall	0.781	0.892	0.442	0.841	0.600	0.590	0.980
		±0.014	±0.010	±0.020	±0.017	±0.019	±0.019	±0.006
	Precision	0.723	0.821	0.554	0.834	0.621	0.587	0.979
		±0.015	±0.013	±0.020	±0.017	±0.019	±0.019	±0.006
	F1 score	0.751	0.855	0.492	0.838	0.610	0.586	0.980
k-NN	Recall	0.673	0.895	0.311	0.819	0.490	0.440	0.944
		±0.015	±0.010	±0.018	±0.018	±0.020	±0.019	±0.010
	Precision	0.636	0.824	0.447	0.826	0.504	0.463	0.772
		±0.016	±0.013	±0.020	±0.017	±0.020	±0.019	±0.018
	F1 score	0.654	0.858	0.367	0.822	0.498	0.450	0.849

## Discussion

The best way to separate the training and validation datasets, would be “subject-wise” cross-validation. In this study, however, it was not possible to obtain evenly distributed datasets in each data fold because the individual subjects each produce a different number of data samples for a given activity. While the collecting data for each activity, a data block was created to overcome this imbalance in the size of the dataset. All data samples for an activity for a given subject was appended behind the previous subject’s data samples in a single row. This is how the datablock was formed. Each subject’s data samples were cut and augmented, but not shuffled. Not shuffling the data samples could minimize the probability of overlap between training and validation datasets. Subsequently, the data block was split into 10 evenly sized datasets. The 10-fold dataset groups for the 10 physical activities were then fed into the CNN for training, and the algorithm performance was assessed with 10-fold cross-validation.

Data augmentation is one of the most commonly used methods when data is limited, especially in deep learning. Due to the characteristics of deep learning, a large amount of input data is required. Augmented data is data that has been slightly modified from the original data for data diversity and robustness of the performance. For example, flipping, rotation, scaling, cropping, translation, and adding noise are commonly used in CNNs that manipulate images. In this study, overlapping and rotation were introduced. These modifications increased the number of data samples and made the algorithm more robust. However, these augmentations depend on the original data. This could cause the results to differ when using data samples that are not augmented. This is the limitation of this study.

It is difficult to discriminate various dynamic physical activities using single waist-mounted accelerometer. Previous work (Preece et al., 2009) showed that the results depended on different accelerometer mount positions: at the waist, thigh, ankle, and combinations of these three positions. The results revealed that the waist location incurs the maximum difficulty when classifying physical activities. Despite this, this study considered device-wearing convenience and representative characteristics of the entire body movement for further field application. The inherent disadvantages of the wearing position and short data period (∼2.8 s) were overcome by using the developed CNN algorithm.

Many prior studies (Foerster, Smeja & Fahrenberg, 1999; Karantonis et al., 2006; Veltink et al., 1996; Zhang et al., 2003) have included posture classes utilizing static acceleration values, such as standing, sitting, lying down, and lying prone. This study was instead focused on physical activities when children were in physically active states, not their postures or static states, as these points (determining levels and modes of activity) are essential for preventing excessive weight or obesity in children. The results of this study may provide assistance in addressing these essential points.

This study demonstrated a method of classifying ten physical activities in children. A classification algorithm was developed and utilized for analyzing recorded acceleration signals, and these results demonstrate the algorithm performance. Table 3 shows the activity classes WS, WF, RS, and RF demonstrated relatively low accuracies. The protocol may have caused these low accuracies: movement speeds were self-selected when subjects conducted the above four activities. If these activities had instead been performed on a treadmill or paced at a preset speed (Harrell et al., 2005; Riel et al., 2016), classifications may have been more distinct; however, treadmill speeds do not represent the innate characteristics or real physical activities performed by children in daily life. It was observed that some participants walked at equal speeds during WS and WF activities, whereas others ran too fast during the RS protocol. Additionally, other participants changed their speed over time, even in same activity of WS, WF, RS, and RF. It is possible that this problem is unavoidable within this age range of participants because they cannot perfectly control their movement speeds; instead, revive the movements of their daily life as real during experimental protocols.

Results revealed two questionable characteristics of the stair moving classes: first, both SU and SD accuracies were relatively low; and second, SU class accuracy was lower than that of SD by 12.5%. In general, staircases are connected in a zig-zag formation for travelling up or down. In this study, participants had to “walk” approximately 3∼4 gaits between staircases in the protocol, unavoidably inserting a walking signal between the recorded stair movement activity signals. Although efforts were made to identify and remove these walking signals, this was particularly challenging between stair climbing (SU) signals. Since these walking signals remained in the staircase movement signals, SU and SD accuracies were low relative to other classes, especially SU. As the SD signal had relatively distinctive acceleration peaks along the gravitational axis, walking signal periods could be identified and eliminated to a somewhat greater extent. Conversely, the SU signal had a relatively even peak envelope and very few walking signal periods were eliminated. This also reduced the SD dataset size relative to the SU dataset, as shown in Table 1.

When SU and SD activities were miscategorized, they were most frequently mistaken for JR outputs; similarly, a considerable error in the JR categorization was in the identification of RS, RF, SU, or SD. When subjects performed the jumping rope protocol, some conducted activities such as walking or running with a rotating rope held in two hands rather than jumping with both feet. They were also children who were physically challenged or poor at rope jumping. These cases are considered to have measured JR activities that appeared closer to RS, RF, SU, or SD than the walking classes because jumping actions increase gravitational acceleration, leading the CNN algorithm to select RS, RF, SU, or SD rather than JR.

As shown in Table 3, the non-ambulatory classes (ST, SI, and NA) were classified with high accuracy. Table 4 shows their excellent performance, quantified by indicators. These non-ambulatory classes were related to intermittent activities and were easily classified correctly. These results are predictable given some CNN algorithm characteristics such as the convolution and max pooling processes. Ambulatory classes related to generating continuous and periodic acceleration signals via repetitive movements, such as walking and running, have relatively similar signal appearances, unlike the above intermittent activity-related classes. These similarities could explain some class confusions, because the convolution and max pooling steps could have compressed the signals until they were indistinguishable. On the other hand, the signal appearances of intermittent activities are relatively apparent, which may enhance the accuracy.

The NA class played the role of a physical activity control group, or negative. If this negative class was not included in the class set, even static signals could be classified as real physical activities. Using the definition of true negative for bi-class cases, NA could be assigned as the negative and all physically active classes could be included as the positive. In this case, the NA class recall could be expressed as the sensitivity of the developed network. By this logic, the developed network specificity would be 99.2%.

Tables 5 and 6 show another aspect of the classification results: the recalculated accuracies after similar activity categories were merged into new integrated classes. The WS and WF, RS and RF, and SU and SD classes were merged into a walking group WX, running group RX, and stair moving group SX, respectively. Thus, the recalculated confusion matrix was shrunk from ten to seven classes. Consequently, this class merger improved the performance indicators because the merged classes were the most frequently mutually confused classes and made up a significant portion of original errors. If this algorithm was embedded into a child activity monitoring device, the merged algorithm case would be most relevant. Decreasing the class number would reduce the number of micro-controller unit executions and also improve accuracy.

The developed algorithm was also compared to conventional algorithms using 128 signal points and a processing window size of 2.8 s. For comparison, similar window size classification examples were sought and cited. A similar prior study (Kühnhausen, Dirk & Schmiedek, 2017) adopted SVM for feature classification, demonstrating an overall accuracy of 87.5% using a general model. However, when this conventional SVM algorithm was used with the current data set, its results were worse than those published in the prior study.

Table 9 displays the poor performance of conventional algorithms. Overall, no class but NA demonstrated good results, revealing these conventional algorithms had insufficient power for classifying the ten individual classes in the original dataset. The developed CNN algorithm (see Table 4), conversely, demonstrated better performance especially in recognition of WF, SU, SD, ST, and SI compared to SVM (which is the best among the alternatives). Table 10 shows the performance indicators of the three conventional algorithms for the seven merged classes case. Although these results were improved from those seen in Table 9, the SX class performance was significantly inferior in all three algorithms even after merging. This indicates conventional algorithms find it difficult to distinguish SX from other activities, meaning SX is too difficult a problem to solve with conventional algorithms. The CNN algorithm, however, shows improved performance in the case of seven merged classes (see Table 7); however, the performance was not as good as that of WX and RX. These facts revealed the dataset used in this study required complex classification and the developed CNN algorithm was superior for this task relative to conventional SVM algorithms.

Generally, the health benefits of physical activity are maximized when the activity is performed in bouts of moderate-to-vigorous intensity (World Health Organization, 2010). However, a recent study (Robson & Janssen, 2015) tells that sporadic moderate-to-vigorous physical activity (MVPA) embedded with bouts of light-intensity physical activity is also healthful. On the basis of this, the developed algorithm could provide not only bouts of MVPA but also embedded MVPA, which occurs frequently for children, and could be used by the caregivers attentive to the activities in the daily life of their children. Furthermore, additional work on this algorithm may be used to infer high-level activities for daily life inferences. For example, if patterns of sports such as soccer or basketball were analyzed, the activity monitor could identify users playing soccer, rather than simply combining basic physical activities such as walking, running, and jumping.

## Conclusions

In this study, the classification of physical activity in children was performed. This study involved algorithm development, participant recruitment, IRB approval, custom-design of a data acquisition module, and data collection. The developed algorithm distinguished physical activities with improved time resolution using short-time acceleration signals from the physical activities performed by children. This algorithm could be applied to real-time applications due to its simplicity.

When the developed CNN algorithm classified the original ten different activity classes, acceptably accurate results were not achieved in some ambulatory related classes; however, class coalescence lead to improved results. Devices and algorithms better able to differentiate between multiple activity classes are able to obtain more information from everyday life; however, merged classes are better suited to embedded systems. Preventing and managing overweight and obesity conditions is more important at young ages, because obese children have higher chances of obesity as adults relative to normal children. Although many devices have been developed to address the issue of weight gain and obesity, such as wrist-worn type accessories and smartphone with software applications, they are unfortunately not applicable to children. The activity monitor proposed in this study could, however, help children substantially by recording how much and long they were physically active, as well as what types of activities filled their active periods.

##  Supplemental Information

10.7717/peerj.5764/supp-1 Supplemental Information 1 Feature table for conventional algorithm comparison (vs CNN) This file is featured from data set of CNN.
- Column titles are 12 features
- Each cell of rows are featured data from individual 2.8 s data (window) from the data set.

10.7717/peerj.5764/supp-2 Supplemental Information 2 The dataset for CNN learning (filtered, cut, augmented from raw data for class 1 ∼5) The zip file contains .mat file which is available for Matlab program. The data set was expressed with ”cell” type so that .xlsx file format was not appropriate for that.
- The 1st layer (10 ×2 cell): Column 1 means target (class), Column 2 means the number of samples.
- The 2nd layer (each cell of Column 2 in the 1st layer/1 ×N (the number of samples of the target) cell): Single sample data of 128 × 1 × 3 (128 ×3).
- The 3rd layer (each single sample data): Columns are axis *x*, *y*, *z* and rows are 128 data point.

10.7717/peerj.5764/supp-3 Supplemental Information 3 The dataset for CNN learning (filtered, cut, augmented from raw data for class 6 ∼10) The zip file contains .mat file which is available for Matlab program.The data set was expressed with ”cell” type so that .xlsx file format was not appropriate for that.
- The 1st layer ( 10 × 2 cell): Column 1 means target (class), Column 2 means the number of samples.
- The 2nd layer (each cell of Column 2 in the 1st layer/1 ×N (the number of samples of the target) cell): Single sample data of 128 × 1 × 3 (128 ×3).
- The 3rd layer (each single sample data): Columns are axis *x*, *y*, *z* and rows are 128 data point.

## Additional Information and Declarations

### Data Availability

The following information was supplied regarding data availability: The raw data are supplied in the Supplemental Files.
